# Abnormal Electroretinogram after Kir7.1 Channel Suppression Suggests Role in Retinal Electrophysiology

**DOI:** 10.1038/s41598-017-11034-1

**Published:** 2017-09-06

**Authors:** Pawan K. Shahi, Xinling Liu, Bryce Aul, Andrea Moyer, Akshita Pattnaik, Jerod Denton, De-Ann M. Pillers, Bikash R. Pattnaik

**Affiliations:** 1 Department of Pediatrics, University of Wisconsin-Madison, Madison, USA; 2 Department of Medical Genetics, University of Wisconsin-Madison, Madison, USA; 3 Department of Ophthalmology and Visual Sciences, University of Wisconsin-Madison, Madison, USA; 4 Department of Pharmacology, Vanderbilt University, Madison, USA; 5 McPherson Eye Research Institute, University of Wisconsin-Madison, Madison, USA

## Abstract

The KCNJ13 gene encodes the inwardly rectifying potassium channel, Kir7.1. Mutations in this gene cause childhood blindness, in which the a- and b-wave responses of electroretinogram (ERG) are abolished. The ERG a-wave is the light-induced hyperpolarization of retinal photoreceptors, and the b-wave is the depolarization of ON-bipolar cells. The Kir7.1 channel is localized to the apical aspects of retinal pigment epithelium (RPE) cells and contributes to a delayed c-wave response. We sought to understand why a defect in an RPE ion-channel result in abnormal electrophysiology at the level of the retinal neurons. We have established the expression of Kir7.1 channels in the mouse RPE. ERGs recorded after mice Kir7.1 suppression by shRNA, or by blocking with VU590, showed reduced a-, b- and c-wave amplitudes. In contrast, the Kir7.1 blocker had no effect on the *ex-vivo* isolated mouse retina ERG where the RPE is not attached to the isolated retina preparation. Finally, we confirmed the specificity of VU590 action by inhibition of native mouse RPE Kir7.1 current in patch-clamp experiment. We propose that mutant RPE Kir7.1 channels contribute directly to the abnormal ERG associated with blindness via alterations in sub-retinal space K^+^ homeostasis in the vicinity of the photoreceptor outer segment.

## Introduction

Leber congenital amaurosis (LCA), a frequent cause of pediatric blindness, is a rare congenital retinal dystrophy that leads to severe visual impairment by mid to late adulthood. Clinical features of LCA include macular pigmentation, nystagmus and an abnormal electroretinogram (ERG)^1–3^. Mutations in 17 genes, including *KCNJ13* which encodes for the inwardly rectifying potassium channel Kir7.1, cause LCA^4–7^. Mutations of KCNJ13 also cause the allelic disorder Snowflake vitreoretinal degeneration (SVD)^5, 8, 9^. Like LCA, SVD patients show a reduced or non-detectible ERG^10^. We have reported a nonsense mutation in the *KCNJ13* gene that results in a truncated and non-functional protein, and leads to vision loss^6^.

Ion channels are proteins with a central pore that are embedded in the lipid bilayer of the plasma membrane. As a group, potassium (K) channels are responsible for maintaining the resting membrane potential of the cell^11^. The subfamily of inwardly rectifying K^+^ channels (Kirs) are tetrameric structures with two transmembrane segments and a pore between them that conducts K^+^ ions across the cell membrane^12, 13^. Kir7.1 channels are weak inwardly rectifying channels that facilitate efflux of K^+^ from cells within the physiological voltage range, thereby contributing to potassium homeostasis of the cellular environment^14^.

The Kir7.1 channel is one of the most recently described members of the Kir channel super-family, and is expressed in brain, nephron, small intestine, and stomach, in addition to the RPE^15–18^. Kir7.1 expression is found on the apical processes (ciliary structures interdigitating with the photoreceptor outer segment) of RPE cells^9, 19^.

The RPE shares a tight sub-retinal space with the photoreceptors (PR)^20^. Light exposure increases cGMP hydrolysis rate, leading to decreased membrane conductance and PR membrane hyperpolarization. The ERG a-wave of the dark-adapted retina is a reflection of photoreceptor hyperpolarization in response to light^21^. Subsequently, a decrease in the release of glutamate from PR synaptic terminals deactivates bipolar cell metabotropic glutamate receptor (mGluR6) to depolarize the ON-bipolar cells as shown by the b-wave^22^. At higher light intensities, the slow c-wave is generated as a result of depolarization of the RPE and glial cells^23^. The sub-retinal space K^+^ concentration drops from 5 to 2 mM at the onset of light in a dark-adapted retina. Kir7.1 channel mediate K^+^ efflux from the RPE cells to the sub-retinal space that offset this transient decrease. The slowly developing c-wave response of the RPE cell demonstrates this overall change in the sub-retinal K^+^ concentration evoked by light^19^.

In the present study, we tested our hypothesis that the ERG abnormality common to LCA and SVD is the result of a non-functional Kir7.1 channel that affects sub-retinal K^+^ homeostasis. We blocked Kir7.1 expression and activity by using shRNA. VU590, a small molecule pharmacological blocker of the Kir7.1 channel, inhibits native Kir7.1 channel in the uterus^24, 25^. We investigated the physiological response of inhibiting the RPE Kir7.1 channels by injecting VU590 into the eyes of C57BL/6 mice, in addition to showing its effect on the isolated retina a- and b-waves by trans-retinal *ex vivo* ERG recording. Lastly, whole-cell patch-clamp recordings of freshly isolated RPE cells, in conjunction with the biochemical analysis of Kir7.1 protein expression, confirmed that Kir7.1 contributes to the normal mouse ERG.

## Results

### Kir7.1 protein is expressed in RPE

**Figure 1:**
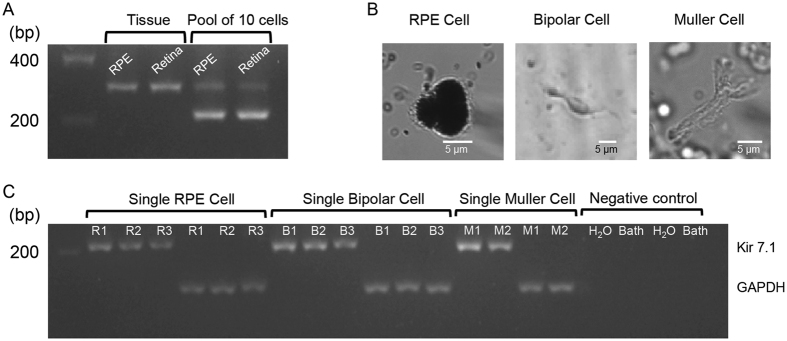
mRNA expression of Kir7.1 in RPE and retina. (**A**) Gel electrophoresis image illustrates the expression of Kir7.1 mRNA in both RPE and retinal tissue, as well as in pools of 10 isolated cells. Complete gel image is included as Supplemental Fig. 1A. (**B**) Images of representative single RPE, bipolar, and Müller glial cell used for single-cell RNA extraction. (**C**) mRNA expression for Kir7.1 and GAPDH in RPE cells (R1–R3), bipolar cells (B1–B3), and Müller glial cells (M1-M2) along with negative control water and perfusion bath solution. A Supplemental Fig. 1C is included showing full gel image.

We first determined kcnj13 transcript expression in separated RPE-choroid and retina preparations. Both samples showed the presence of a 308 bp Kcnj13 PCR product in mRNA isolated from tissue samples (Fig. 1A lanes 2 and 3). To overcome any possible contamination of cells from either the RPE layer or retinal layer, we isolated mRNA from a pool of 10 RPE cells or 10 retinal neurons. We detected a 230 bp Kcnj13 PCR product in both cell types (Fig. 1A lanes 4 and 5). The freshly isolated single RPE cells were identified based on the appearance of a figure-8 shaped pigmented cell with visible apical processes (Fig. 1B left panel). The retina neurons were morphologically identified as either bipolar or Müller cells (Fig. 1B). Kcnj13 bands of 230 bp were also detected in individual cells along with bands for the housekeeping gene Gapdh 124 bp (Fig. 1C).

**Figure 2:**
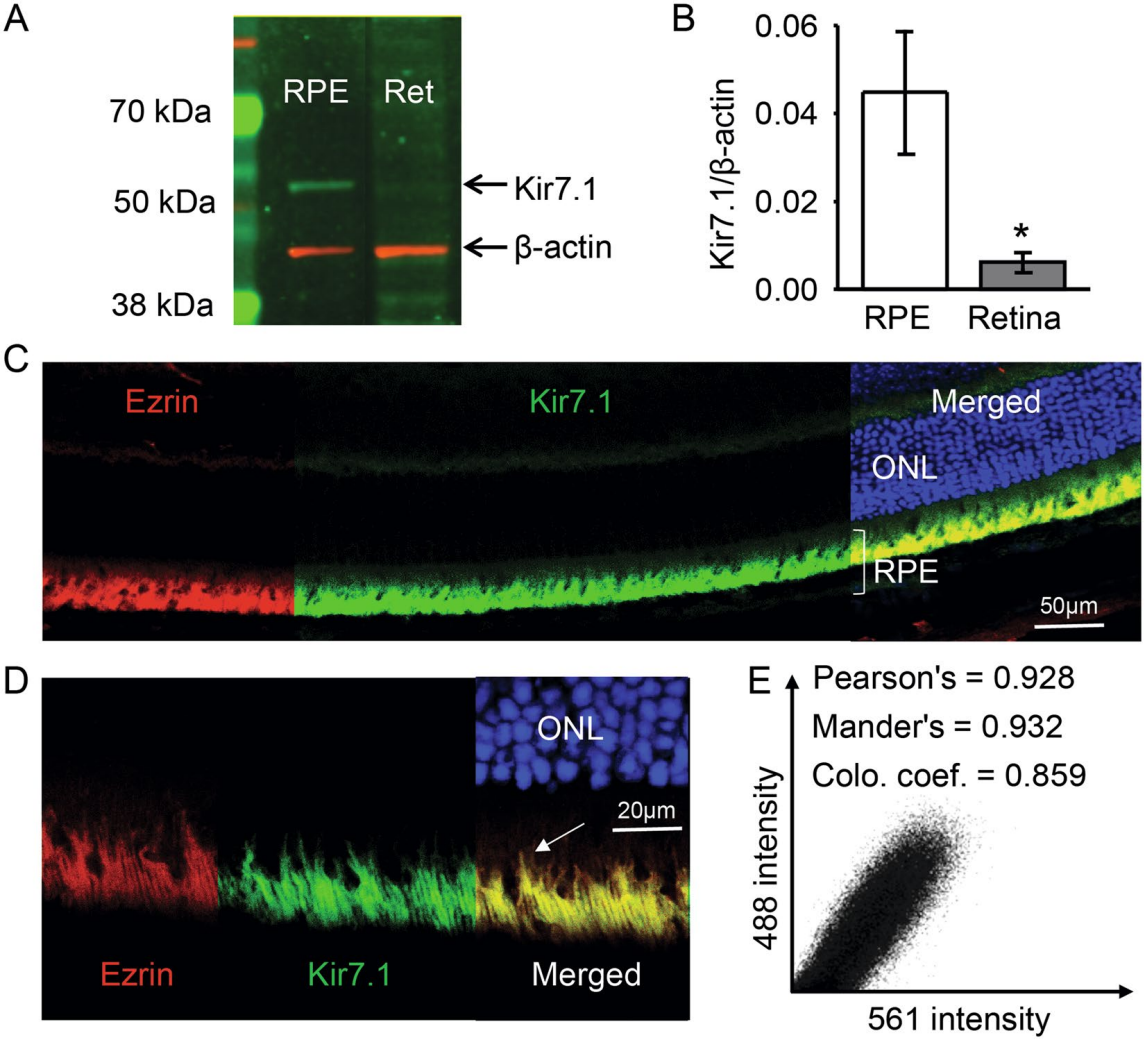
Kir7.1 protein expression. (**A**) Protein expression of Kir7.1 in RPE and retinal tissue detected by Western blot analysis. Complete Western blot image is included as Supplemental Fig. 2A. (**B**) Bar graph showing the relative expression of Kir7.1 protein in the RPE and the retina by densitometry expressed as a Kir7.1/b-actin ratio. (**C**) Immunohistochemistry against Kir7.1 and Ezrin; ezrin (red) labels the microvilli of the RPE cells and Kir7.1 (green) co-localizes with ezrin confirming its presence in the apical processes of the RPE cell. Outer nuclear layer (ONL) is stained with DAPI (blue). Scale bar is indicated. A video is included as a Supplemental data. (**D**) Higher magnification image of the RPE layer shows ezrin expression (red) in apical processes extending towards the retina. Kir7.1 (green) staining also appeared in apical membrane extensions with co-localization of both proteins (yellow) in long apical processes (arrow). Scale bar is included. (**E**) A quantitative distribution plot representation of signals acquired in green (488) vs. red (594).

We also measured protein expression in both RPE cells and retina, and compared gene expression levels with protein abundance. Using a Kir7.1 commercial antibody for Western blotting, we observed a 52 kDa Kir7.1 strong protein band in the RPE, with only a faint band noted in the neural retina, as shown in Fig. 2A. Quantification of protein relative to expression of the housekeeping gene b-actin showed an approximately 4-fold relative abundance of the protein in the RPE as compared to the neural retina (Fig. 2B). Immunofluorescent staining of mouse eye cryosections showed that RPE cells stained for both ezrin (Fig. 2C, red) and Kir7.1 (Fig. 2C, green) protein. When merged with nuclear staining, the color appeared to be yellow thereby indicating the co-localization of ezrin and Kir7.1 to the RPE cell layer (Fig. 2C). A higher magnification image further delineated the exact localization of ezrin and Kir7.1 to the RPE cell apical processes that extend towards retina (Fig. 2D white arrow and Fig. 2E).

### shRNA targeting of Kir7.1 inhibited mRNA expression and reduced ERG

**Figure 3:**
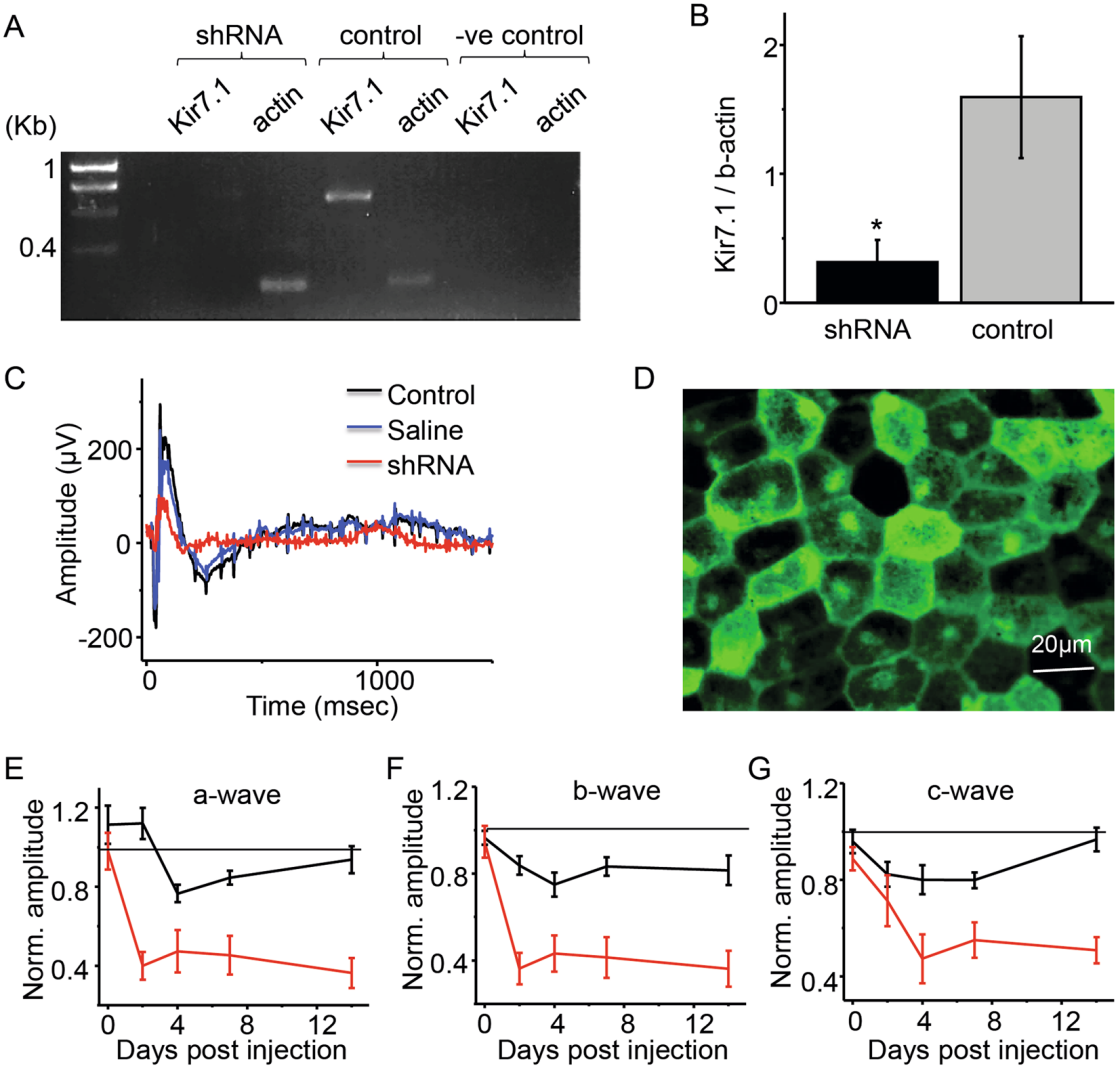
Reduced RPE/retina function after sub-retinal administration of lentivirus containing shRNA for Kir7.1. (**A**) Gel electrophoresis showing that Kir7.1 mRNA expression is reduced after Kir7.1 shRNA injection when compared to the un-injected contralateral eye. Full image of the gel is included as Supplemental Fig. 3A. **(B**) Expression of Kir7.1/b-actin ratio in shRNA injected and un-injected eye (part A) represented in a bar graph. (**C**) Representative scotopic traces comparing the shRNA-injected mice 14 d post injection, as well as control and PBS-injected mice. (**D**) Fluorescent image of live RPE sheet of cells displaying the expression of GFP that is fused with the shRNA Kir7.1. (**E**) Normalized a-wave and (**F**) normalized b-wave comparing the shRNA injected eye with the PBS injected eye at 30 cd.s/m^2^ at 0, 2, 4, 7, and 14 d post-injection. (**G**) Comparison of c-wave for eyes injected with Kir7.1 shRNA and PBS at 0, 2, 4, 7 and 14 d post injection. Dotted line represents the control-normalized response from non-injected eyes.

After demonstrating that Kir7.1 protein is primarily expressed in RPE cells, we sought to determine whether the RPE Kir7.1 channel contributes to the ERG. We delivered a specific shRNA lentiviral particle to the mouse vitreal chamber by micro injection to inhibit Kcnj13 gene expression. Seven days after shRNA injection, eyes were harvested and RPE cells isolated. PCR amplification of Kcnj13 in RPE cells did not reveal a product from the shRNA injected mouse eye (Fig. 3A Lanes 3), whereas in the contralateral eye that received saline injection, a 653 bp band corresponding to the Kcnj13 transcript was present (Fig. 3A Lane 5). Housekeeping gene ß-actin transcript was detected as a 248 bp band in both samples (Fig. 3A Lanes 4 &6). We did not detect either Kir7.1 or actin in negative control experiments (Fig. 3A Lanes 7 & 8). Kir7.1 shRNA inhibition was able to reduce Kcnj13 mRNA expression by an average of 73.62 ± 14.47% (Fig. 3B).

We have previously reported that short term inhibition of Kir7.1 by shRNA suppresses the mouse ERG but not the use of scrambled shRNA^6^. To determine the specificity of shRNA action, electroretinography was performed on mice at 2, 4, 7, and 14 d post-injection of Kir7.1 shRNA. We did not find any significant difference in the amplitude of the ERG a-, b- and c-waves between saline injected versus control eye. All ERG findings are normalized to the contralateral non-injected eye of the same mouse. The normalized value 14 d post injection for the a-wave was 0.94 ± 0.02 (P = 0.04), the b-wave was 0.82 ± 0.03 (P = 0.093) and the c-wave was 0.97 ± 0.01 (P = 0.52). In contrast, there was a significant reduction in the a-, b- and c-wave amplitudes after shRNA-mediated loss of Kir7.1 expression. When normalized against the saline injected eyes, the amplitudes of the a-, b- and c-waves were observed as 0.36 ± 0.02 (P = 0.0003), 0.36 ± 0.03 (P = 0.0028) and 0.51 ± 0.01 (P = 0.0001), respectively (Fig. 3C,E,F & G). A slight decrease in the a-, b- and c-waves was observed in saline-injected mouse eyes 2 and 4 days post-injection, but they completely recovered after 7 days, unlike the Kir7.1-silenced eyes. After 14 days of scrambled shRNA or Kir7.1 shRNA injection, c-wave amplitude was reduced only due to Kir7.1 silencing (Supplemental Fig. 3B,C). We also tested for functional Kir7.1 channel in isolated mouse RPE cells through whole-cell patch-clamp measurements. Results (Supplemental Fig. 3D,E) show that scrambled shRNA receiving RPE had a huge inward Kir7.1 current measured as Rb+ conductance but not the Kir7.1 silenced RPE cells. So we conclude that Kir7.1 shRNA specifically knocks down RPE Kir7.1 channel in mice.

The lentivirus carrying the shRNA specific to Kir7.1 was also fused with GFP protein in order to confirm transduction by the virus and shRNA knock-down of Kir7.1 protein production in the RPE. Six mice were injected and all six mice demonstrated ERG waveform changes. We sacrificed each of the six mice with reduced ERG waveforms at 14 d post injection, and the eyes were enucleated. The retina was removed and the flat mount of the RPE was obtained. GFP fluorescence in cuboidal RPE cells was detected, thereby confirming the targeted delivery of the shRNA (Fig. 3D).

### Pharmacological inhibition of Kir7.1 channel also alters ERG

**Figure 4:**
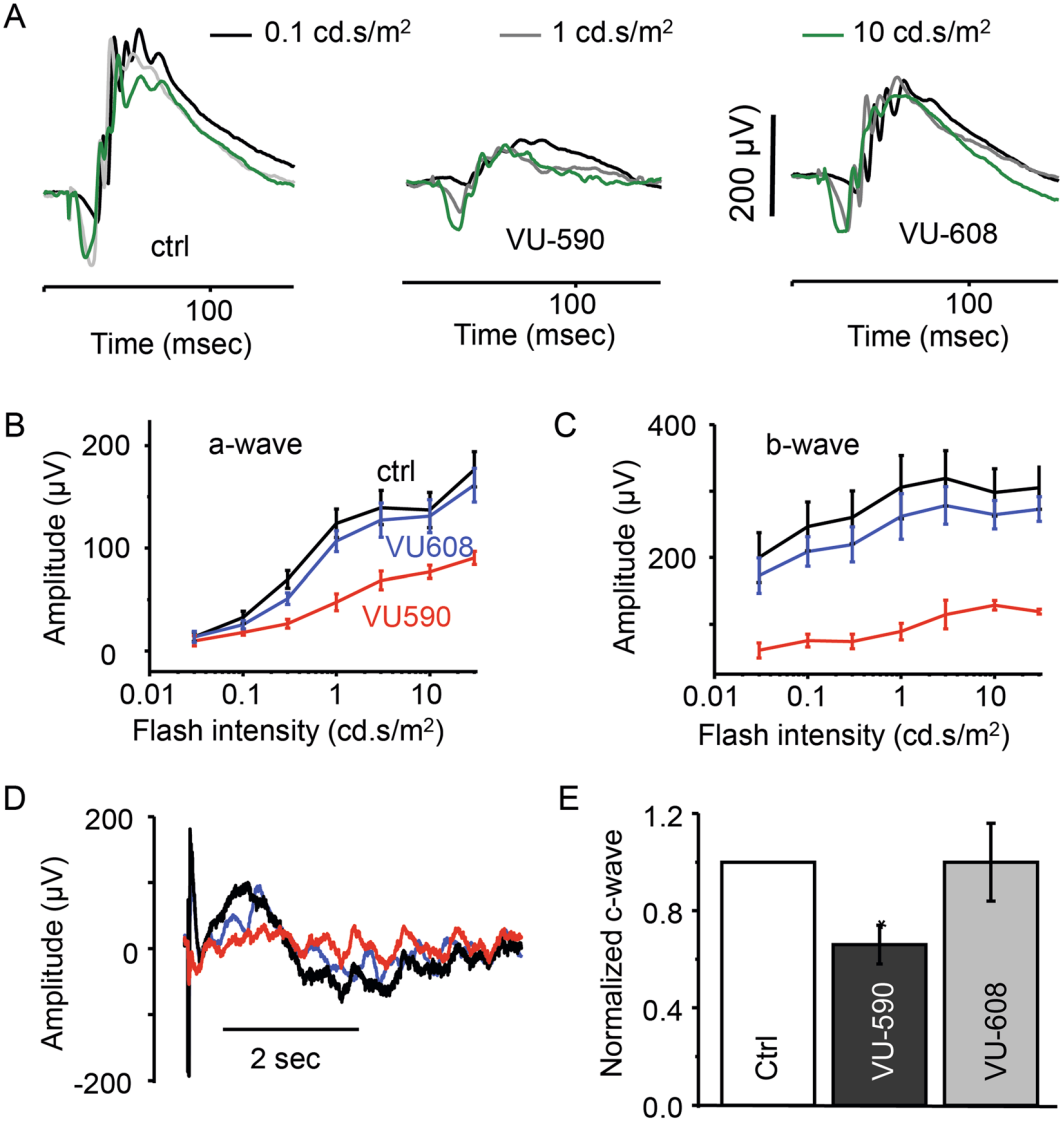
Kir7.1 channel blocker VU590 inhibits c-wave originating from RPE cell. (**A**) Representation of the scotopic traces at different intensities from control mice and mice injected with VU590 or VU-608, an inactive analogue of VU-590. (**B**) Amplitude of a-wave and (**C**) b-wave after VU590 injection (red) compared with the VU608 injected eye (blue) and the control (black) saline injected eye. (**D**) Scotopic ERG trace representing the reduction of c-wave after the injection of VU590 but not with VU608 when compare with control. (**E**) Graphical representation of the c-wave reduction after Kir7.1 channel inhibition. Data is mean ± SEM and *P < 0.005.

VU590, a small molecule Kir7.1 channel inhibitor^26^ was injected into the vitreous of one eye (50 µM solution 2 µl) and 2 µl of saline was injected into the contralateral eye as an experimental control. Mice were dark-adapted for 3 hours immediately after injection and prior to performing electroretinography. We observed a decrease in the a-, b- and c-wave amplitudes in the VU590 injected eye, with normal ERG recordings in the saline-injected control eye. Figure 4A demonstrates the unaffected a- and b-wave amplitudes in response to 0.1, 1 and 10 cd.s/m^2^ flash intensities in saline-injected control eyes (Fig. 4A left panel). At the same flash intensities, a profound reduction in the a- and b-waves was observed in VU590-injected mice (Fig. 4A middle panel, B, and C). The normalized values for the a- and b-waves when compared to the contralateral saline-injected eyes were 0.65 ± 0.04 (P = 0.02) and 0.48 ± 0.07 (P = 0.02, n = 4), respectively. We further tested the specificity of VU590 action by injecting 50 µM of VU608, an inactive analogue of VU590, as a control for any nonspecific effects that could be attributed to the small molecule. The ERG recordings showed normal results, with both a- and b- wave amplitudes similar to those of the control eyes (Fig. 4A right panel, B, and C), with the normalized values for the a- and b-wave amplitudes being 0.90 ± 0.04 (P = 0.11) and 0.88 ± 0.07 (P = 0.16, n = 6), respectively.

We also tested the effect of VU590 and VU608 on the ERG c-wave. We measured a significantly reduced c-wave in the VU590 injected eye when compared to saline injected eyes. There was no effect on the c-wave by VU608 (Fig. 4D). The normalized values for VU590 and VU608 in comparison to control were 0.66 ± 0.04 (P = 0.02, n = 4) and 0.94 ± 0.05 (P = 0.38, n = 6), respectively (Fig. 4E). VU590 reduced the amplitude of the a-wave by 30%, the b-wave by more than 50% and the c-wave by 35%. We speculate that the 35% attenuation of the c-wave generated by RPE cells was sufficient to alter the physiological properties of photoreceptors, thereby leading to the decrease in the amplitude of a- and b- waves.

### Retina structure appears normal after injection

**Figure 5:**
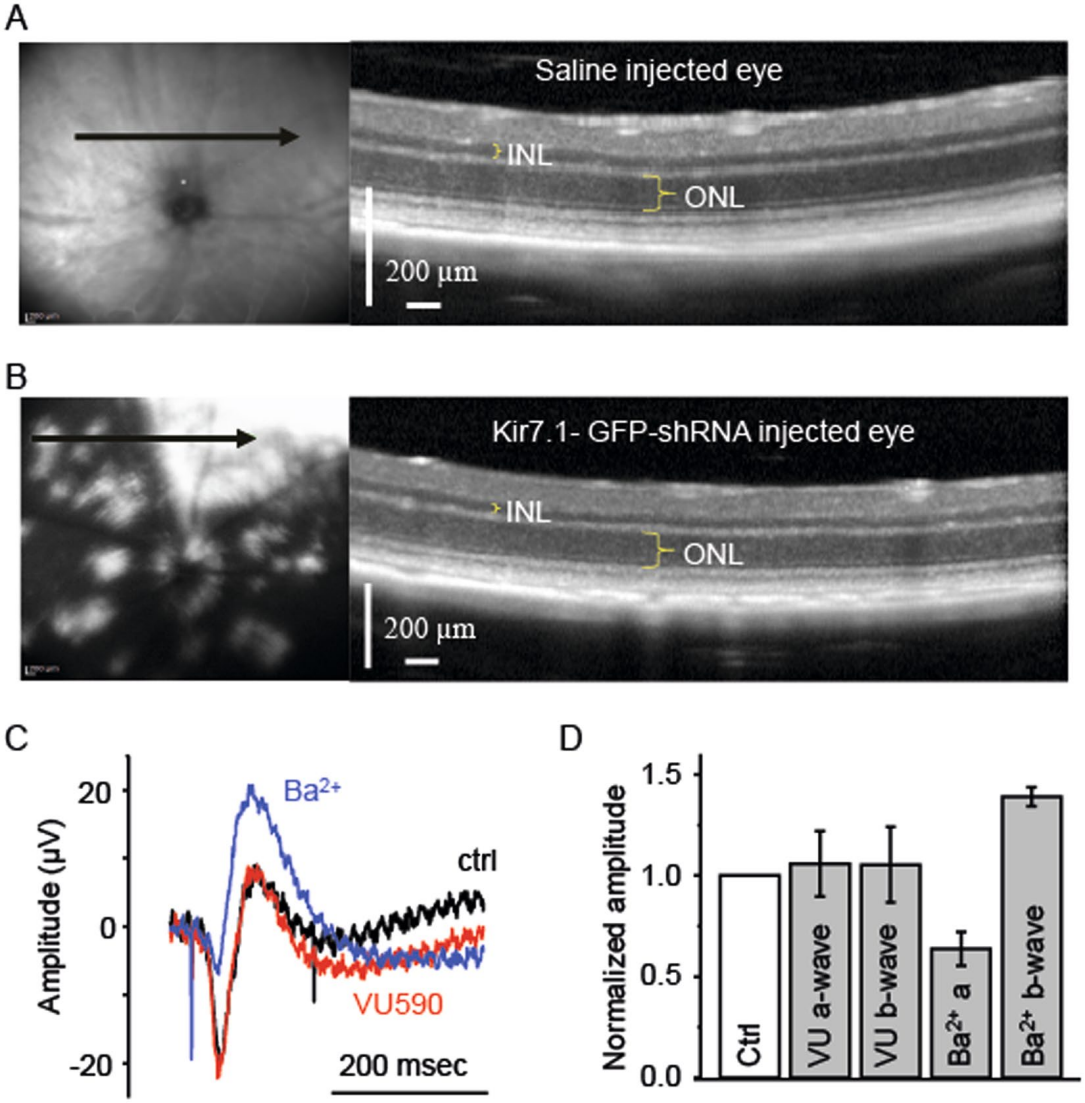
Optical Coherence Tomography imaging of saline (**A**) and shRNA (**B**) injected eyes to visualize the structural integrity of the RPE and retina. In both panels A and B, the fundus photo shown in the left panel indicates the orientation (arrow) of the cross-sectional OCT. The scanned images with the outer nuclear (ONL) and inner nuclear (INL) layers is highlighted in the panel on the right. In image B, GFP fluorescence is evident in the white patches. (**C**) *Ex-vivo* ERG allows recording the response from the retina, only. ERG traces from the retina before (black) and after the treatment with 50 µM VU590 (red) are shown. The proper functioning of the ERG and the retina is confirmed by the treatment with 100 µM Ba^2+^ (blue). (**D**) Bar graph representing the amplitude of a- and b-waves in *ex-vivo* ERG recording after treatment with VU590 and Ba^2+^ are shown as compared to control.

We next confirmed that the ERG changes in our experiments were not simply due to structural changes in the retina or RPE from physical damage by the injections. We obtained *in vivo* OCT images from eyes 14 d post injection with saline or shRNA, and after the ERG was performed. As shown in Fig. 5A, the OCT scan location for the saline-injected eye shown on the left panel is superior to the optic nerve head (Fig. 5A left panel green arrow). The OCT image showed normal thicknesses for the inner nuclear layer (INL), outer nuclear layer (ONL) and retina (Fig. 5A right panel). The reflective RPE layer was preserved. In the shRNA injected eye, we noticed GFP fluorescence in patches (Fig. 5B left panel). Other than the GFP distribution in the shRNA injected eye, we did not see any difference in layer thickness between saline and shRNA injected OCT images. This was also confirmed by counting of ONL cells after H&E staining of fixed retina tissue (Supplemental Fig. 5A–C).

### Kir7.1 inhibitor VU590 has no effect on isolated retinal ERG

To ensure that VU590 had no direct effect on the retina nor that it directly affected retina Kir channels, we tested VU590 using an *ex vivo* isolated retina preparation in which the RPE was not present. Transretinal recordings showed normal a- and b-waves (Fig. 5C, black trace), and upon exposure to 50 µM VU590, the a- and b-waves were unchanged (Fig. 5C, red trace). We used 100 µM Ba^2+^ (Fig. 5C, blue trace) as an experimental control as it has been shown to increase the amplitude of the b-wave^27, 28^. As shown in Fig. 5D, the amplitude of VU590 treated a- and b-waves normalized to no-drug control was identical to control values, measuring 1.101 ± 0.071 (P = 0.17, n = 5) and 1.097 ± 0.09 fold (P = 0.977, n = 5), respectively. Treatment of the retina with Ba^2+^ resulted in a decrease in the a-wave to 0.6 ± 0.08 fold (P = 0.001, n = 6), and increased the amplitude of the b-wave by 1.39 ± 0.5 fold (P = 0.006, n = 6) when compared with control retina responses. Taken together, our results indicate that VU590 inhibition of the RPE Kir7.1 channel had a direct effect in reducing the mouse ERG a- and b-wave amplitudes.

### VU590 specifically inhibits RPE Kir7.1

**Figure 6:**
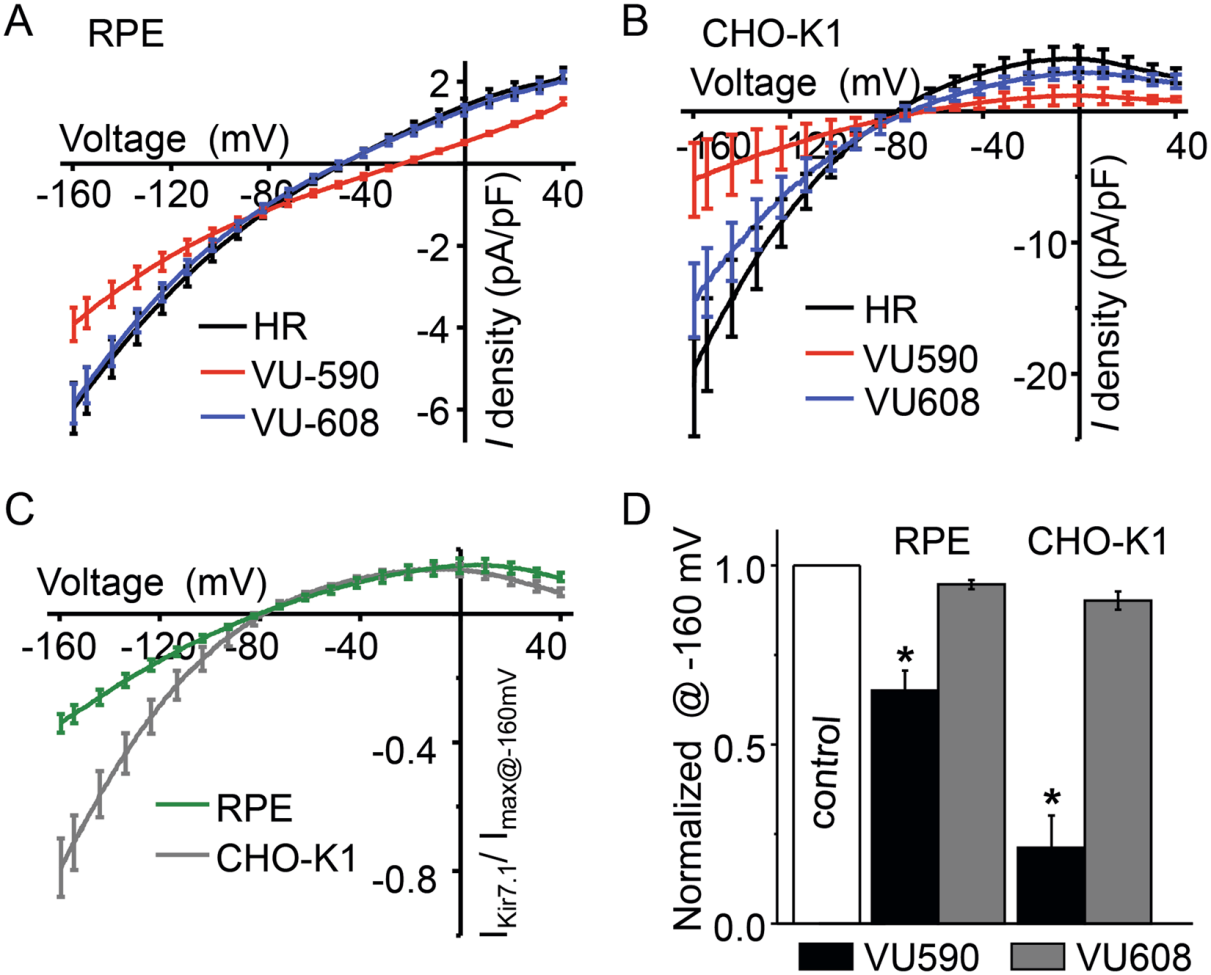
Inhibition of Kir7.1 current by VU590. Representative traces of whole cell currents recorded from single isolated RPE cells from mouse (**A**) and CHO-K1 cells (**B**) overexpressing the Kir7.1 channel, respectively. Whole cell currents were recorded by voltage ramping from +40 mV to −160 mV. The traces represent current density, which is the whole cell current normalized for the cell capacitance. In both cell types, the normal baseline Kir7.1 current is recorded prior to cells being treated with 50 µM VU590. The current is reduced after treatment with VU590, but is not affected by the treatment with 50 µM VU608. (**C**) and (**D**) The normalized Kir7.1 current at −160 mV is compared before, and after, treatment with VU590 or VU608.

As a third line of evidence, we used whole cell recording of isolated RPE cells to determine the effect of VU590 (50 µM) on RPE cell physiology. We applied voltage ramp pulses from −160 to +40 mV every 10 seconds to record the Kir7.1 current amplitude. Average current-voltage plots in control solution (Fig. 6A, black trace HR), in the presence of VU590 (Fig. 6A, red trace), and in the presence of 50 µM VU608 (Fig. 6A, blue trace) show that both inward and outward currents were inhibited by VU590, but not by VU608. Inward current density in the saline control RPE cells measured −5.97 ± 0.6 pA/pF (n = 12) at −160mV. Upon treatment with VU590 (50 µM), the inward current was reduced to −3.92 ± 0.4 pA/pF (p = 0.001, n = 6), which is approximately 2 pA/pF less than the control recording (Fig. 6A). When the cells were exposed to VU608, the inward current at −160 mV measured −5.85 ± 0.4 pA/pF (P = 0.056, n = 6), which was no different than the control response of −5.97 ± 0.6 pA/pF. Using heterologous expression of Kir7.1 in CHO-K1 cells, we further showed that VU590 inhibited the Kir7.1 current (Fig. 6B, red trace), whereas VU608 did not (Fig. 6B, blue trace). Kir7.1 inward current measured at −160 mV was 19.54 ± 3.9 pA/pF in the control solution, and was reduced by VU590 to 5.23 ± 2.8 pA/pF (P = 0.0049, n = 4). In contrast, the inward current was 14.5 ± 2.8 pA/pF (P = 0.07, n = 4) when Kir7.1 channels were exposed to VU608. In Fig. 6C, we show the current-voltage plot for VU590-sensitive component (by subtracting VU590 current from current in presence of control solution) normalized to maximum whole cell current in RPE cells (Fig. 6C, green trace) and CHO-K1 cells (Fig. 6C, grey trace). Current-voltage plots clearly illustrate an inwardly-rectifying Kir7.1 channel with a zero-current potential of −80 mV, very close to the measured E_K_ of −88 mV. On average, VU590 blocked 40% of RPE whole-cell current and 80% of Kir7.1 current (Fig. 6D). VU608 had no significant effect on either the RPE whole cell current or the Kir7.1 current. In summary, VU590 specifically inhibited RPE cell Kir7.1 current, whereas its inactive isomer VU608 had no significant effect.

## Discussion

In this study to delineate the cause of blindness associated with KCNJ13 mutations, we have made several important discoveries that point to an underlying mechanism. We have shown that the transcript for Kir7.1 is present in both RPE cells as well as the neural retina in mice. However, Kir7.1 protein was only localized to mouse RPE cell apical processes. Either suppression of Kir7.1 expression using shRNA, or short term via Kir7.1 pharmacological inhibitor injections to the mouse eye, resulted in a severely reduced amplitude ERG. Our study has several strengths. Firstly, we demonstrate that there is a discrepancy in mRNA and protein expression at a cellular level between RPE and retinal cells. Secondly, we confirmed that the ERG phenotype in KCNJ13-related blindness originated at the RPE cells and is not due to a defective retina. Thus, we demonstrate the contribution of RPE Kir7.1 to the ERG, as was speculated by Wu *et al*.^29^.

### Kir7.1 transcript and protein expression

Kir7.1 transcript is present in rat^30^, porcine^31^, bovine^19, 32^, rhesus^33^, and human^33^ RPE cells. In bovine, rhesus and human, Kir7.1 transcript was present in both RPE and retina. It is possible that there is a species-specific expression that results in this inconsistency. After separating mouse RPE and neural retina, we found almost similar expression of Kir7.1 in both tissues (Fig. 1A). Using single cell PCR amplification from mRNA harvested from either individual RPE or retina neurons, there was a consistent expression of Kir7.1 mRNA in both cell types (Fig. 1B,C). In human, Kir7.1 is also expressed in a variety of tissues, including the small intestine, brain, and kidney^17, 34^.

We detected Kir7.1 protein in RPE cells. Quantitative expression analysis compared to control protein also showed a faint Kir7.1 band in the retina preparation (Fig. 2B). This may simply be a nonspecific interaction with the antibody (as has been suggested earlier by Yang *et al*. for bovine tissue), or contamination of the retina with RPE apical processes^19^. A low abundance of the protein in the neural retina may also explain these results. Whether there are sufficient amounts of protein expressed in the neural retina to contribute to retinal function is unclear, but our data have not demonstrated that it plays a significant role in retinal electrophysiology.

### Kir7.1 channels are confined to RPE apical processes

We have shown that Kir7.1 is localized to the rhesus RPE apical processes, similar to the findings of Yang and colleagues who have shown that Kir7.1 is present in bovine apical processes^9, 19^. Thus, we have resolved prior uncertainties regarding the exact localization of Kir7.1 in the RPE cells^30, 35^. We found that there is immunoreactivity of Kir7.1 in the extending RPE apical processes and that it is not limited to the apical process root structure^32^ (Fig. 2C,D). These long processes extend towards the photoreceptors. Ezrin, which labels RPE apical processes, co-localized with Kir7.1, as previously noted for bovine retina^19^. The photoreceptor outer segments and the RPE apical processes are separated by a very narrow sub-retinal space, and we propose that Kir7.1 may play a crucial role in maintaining sub-retinal space integrity. This is also evident from examination of the tissue after 14 days of Kir7.1 silencing which results in altered photoreceptor inner and outer segment morphology (Supplemental Fig. 5A and B) consistent with CRISPR mediated Kir7.1 knock down. Since we have only studied two weeks after silencing, the structural consequences might not be as severe as the CRISPR mediated knockdown, which we aim to address in the future.

### Alteration of sub-retinal space K^+^ homeostasis affects retina PR function

Both apical Na-KATPase^36, 37^ and Kir7.1^30^, facilitate K^+^ recycling. Within the physiologic membrane potential, K^+^ efflux is mediated by the Kir7.1 channel. In contrast, K^+^ uptake occurs through Na-KATPase in order to keep a sub-retinal space K^+^ concentration of 5 mM during dark conditions. At the onset of a light stimulus, the sub-retinal space K^+^ concentration drops to 2 mM. Dark-light transitions that affect RPE K^+^ conductance are defined based on findings of light-induced loss of extracellular K^+^ and hyperpolarization of cells in a frog eye cup preparation^38^.

Kir7.1 is the major K^+^ conductance channel in the RPE apical membrane where it is expressed at high density and when it is blocked, RPE cells are depolarized^19, 32, 33, 39–41^. Inhibition of Kir7.1 conductance by micro RNA results in reduced apical membrane conductance^42^. Consistent with the regulation of RPE physiology by a light-peak substance, we have shown that the Kir7.1 channel in the RPE can be inhibited by receptor-mediated membrane PIP2 depletion^43, 44^. When we inhibited Kir7.1 channel expression through shRNA or blocked the channel function by VU590, there was a severe reduction in the ERG a, b, and c-waves (Figs 3 and 4) and single cell Kir7.1 current. Similar findings have been shown in mice when Kir7.1 is knocked out by CRISPR-mediated gene editing^35^.

The ERG a-wave reflects the hyperpolarization of photoreceptors due to inhibition of dark circulating current^21^. In normal physiology, Kir7.1 channels are responsible for K^+^ efflux from the RPE^32^. Upon altering Kir7.1 channel function, the dark-light transition of the sub-retinal space homeostasis is altered^45^. Our isolated retina ERG findings showing that Kir7.1 inhibition had no effect on either a- or b-wave, are consistent with this model of sub-retinal space homeostasis. As expected, low concentration Ba^2+^, which is known to inhibit highly sensitive Kir channels (like Kir4.1) in the retina and alter the SlowPIII component, significantly altered the ERG response in our experimental system. Repolarization of RPE cells generates a delayed cornea positive c-wave due to activation of Kir7.1 channels^38^. Upon inhibition of Kir7.1 channel function, either by inhibiting its expression via shRNA, or by blocking the Kir7.1 conductance by the small molecule inhibitor, VU590, reduced the c-wave response in mice.

### Model for the ERG being influenced by Kir7.1

**Figure 7:**
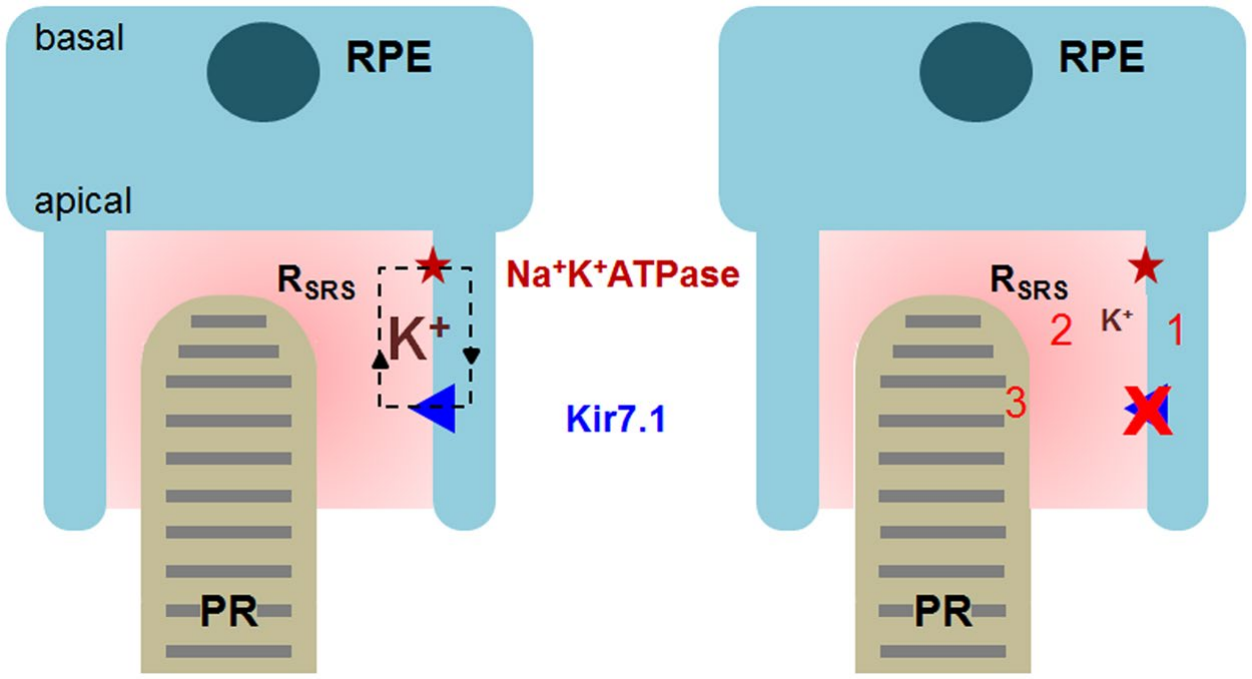
Control of sub-retinal space K^+^ homeostasis by Kir7.1 is crucial for photoreception. On the left is a representation of a normally functioning Kir7.1 channel which is able to maintain normal K^+^ levels. On the right, a reduction in K^+^ in the sub-retinal space due to disease that alters the ERG. The model: 1- Depolarization of RPE, 2- Changes in the sub-retinal space volume and K^+^, and 3- PR ionic conductance. RPE: retinal pigment epithelium, PR: photoreceptor, R_SRS_: sub-retinal space resistance.

We conclude that the ERG is influenced by Kir7.1 activity in the RPE apical processes (Fig. 7). Firstly, RPE cells will be depolarized in the absence of a functional Kir7.1 channel and will affect all other transport properties such as the coupled Na-KATPase. Second, efflux of K^+^ through these channels maintains a K^+^ source for K^+^ recycling and contributes to the dark current, sub-retinal space volume and sub-retinal space homeostasis. Third, the CNG channels in the photoreceptors are permeable to both Na^+^ and K^+^ to facilitate phototransduction. In the absence of K^+^ in the sub-retinal space, the CNG channel function is probably compromised in its ability to uptake K^+^, which is why the ERG is reduced. If we argue that under physiological condition CNG channel mediate Na^+^ and Ca^2+^ conductance, lower K^+^ in the subretinal space due to inactive Kir7.1 channel could increase the driving force for PR outer segment K^+^ flux which might decrease the CNG channel net inward current leading to a decreased a-wave response.

Yang and colleagues determined that Kir2.1, Kir3.4 and Kir7.1 transcripts are all highly expressed in human RPE cells^46^. Other than the potassium conductance noted for Kir7.1, a functional potassium conductance for either Kir2.1 or Kir6.1 has yet to be found in the RPE, whereas in the retina, Raz-Prag and colleagues have shown that Kir2.1 in bipolar cells mediates the corneal-negative ERG^47^. The abundance of Kir4.1 in the end-feet of Müller glial cells contributes to potassium clearance from retina to the vitreous^48^. Collectively, it appears that starting with Kir7.1 in the RPE, followed by retinal Kir2.1 and Kir4.1, the family of Kir channels act to integrate the potassium homeostasis critical for visual information processing.

### Medical implications

Mutations within the KCNJ13 gene cause SVD^8–10, 49^ and LCA16^4–7, 45^. We have found that KCNJ13 mutations result in a dysfunctional Kir7.1 channel. While the SVD mutation likely alters channel regulation by PIP2, LCA16 nonsense mutations result in a truncated protein product that is not able to function as a Kir7.1 channel^6, 9^. We have shown that there is an intrinsic effect of the mutant channel on the potassium conductance of the RPE apical membrane to abolish ERG.

In summary, we have shown that in the RPE, Kir7.1 is confined to the RPE apical processes where it is known to mediate K^+^ efflux. K^+^ conductance in the apical membrane is crucial for both recycling of K^+^ and maintaining a hyperpolarized membrane potential. Precise suppression of Kir7.1 expression by shRNA, or inhibition by the small-molecule inhibitor VU590, abolishes the mouse ERG. This effect is RPE in origin, as VU590 had no effect on the ERG in an isolated retina preparation that does not include the RPE. At light onset, PR outer segment CNG channels shut down, and the ensheathing of the PR outer segment by the RPE apical processes acts to tightly control K^+^ ion flux. In support of our findings, the reduction of the ERG amplitudes after sodium iodate-mediated loss of RPE cells also emphasizes the interaction of the PR outer-segment and RPE apical process as being crucial for shaping the *in vivo* ERG^50^. Our study further demonstrates that even if RPE cells are intact at a morphologic level, functional Kir7.1 channels are required for normal physiology. Inhibition of the mouse RPE Kir7.1 channel also caused a depolarization of the membrane potential. Thus, we propose that the ERG phenotype in blindness caused by mutations in KCNJ13 is due to a loss of the RPE cell ability to control potassium homeostasis around the photoreceptor outer segment where visual transduction originates.

## Materials and Methods

### Ethical statement

The University of Wisconsin –Madison Medical School is accredited by AALAC and all experimental animals were handled in accordance with the animal use protocol approved by the University of Wisconsin School of Medicine and Public Health Animal Care and Use Committee (ACUC). We strictly followed the ARVO statement for the use of animals in ophthalmic and visual research.

### Animals, Anesthesia, and Microinjection

6 wk old C57BL/6 J mice (both male and female) were obtained from a breeding colony at the University of Wisconsin Biotron (Madison, WI). They were maintained under tightly controlled temperature (23 ± 5 °C), humidity (40–50%) and light/dark (12/12 h) cycle conditions in 200 lux light environment. The animals were dark-adapted overnight prior to performing the ERG. The mice were anesthetized with ketamine (80 mg/kg) and xylazine (16 mg/kg). Either 2 µl of shRNA (sc-155912-V with a GFP tag, Santa Cruz Biotechnology Inc., Dallas, TX), or a pharmacological inhibitor was injected into the sub-retinal space or the vitreous, using a UMP3 ultra-micro pump fitted with a NanoFil syringe, and the RPE-KIT (all from World Precision Instruments, Sarasota, FL). Prior to the ERG recordings, the cornea was anesthetized with a drop of 0.5% proparacaine HCl and the pupil was dilated with 1% atropine. Mice were placed on a temperature-regulated heating pad throughout the recording, during the injection, and for recovery purposes to maintain thermal stability.

### Isolation of RPE Cell

Four to six wk old mice were anesthetized and sacrificed by cervical dislocation. The eyes were enucleated and immediately placed in ice-cold sodium-calcium-magnesium-free solution (0Na-CMF: 135 mM NMDG-Cl, 5 mM KCl 10 mM HEPES, 10 mM Glucose, 2 mM EDTA-KOH and pH adjusted to 7.4) and washed × 2. An incision was made in the scleral buckle using a sharp needle. Vannas scissors were used to cut along the scleral buckle to open the eye and the anterior cornea. The iris and lens were discarded. With the use of surgical forceps, the thin layer of the retina was slowly peeled away, leaving intact the RPE layer within the posterior eye cup which was transferred to a 2 ml tube containing 250 µl of the enzymatic solution (0.375 mg/ml Adenosine, 0.3 mg/ml L-Cysteine, 0.25 mg/ml Glutathione, 0.05 mg/ml Taurine, 2.5 µl/ml papain and 5 µl/ml DNAse (0.8 mg/ml-stock) dissolved in 0Na-CMF solution) at 37 °C for 30 min. The reaction was stopped using 0.01% BSA, and the eye cups were washed gently with warm HEPES ringer solution (135 mM NaCl, 5 mM KCl, 10 mM HEPES, 10 mM Glucose, 1.8 mM CaCl_2_, 2 mM MgCl_2_, pH adjusted to 7.4) containing 0.3 mg/ml Glutathione and 0.05 mg/ml Taurine. The cells were dispersed by tapping the tube gently and allowing them to settle.

### RT-PCR and single cell RT-PCR

RPE cells isolated from mice were used to isolate total RNA. cDNA was obtained as discussed below for single cell RT-PCR. We used the mouse Kcnj13 external primer pair and 35 PCR cycles to amplify the PCR product from pooled RPE/retina cells. Denaturing, annealing, and extension began with one cycle at 95 °C for 5 min, followed by 35 cycles at 94 °C for 30 s, 55 °C for 30 s, 72 °C for 30 s, and final extension at 72 °C for 10 min using a ProFlex PCR system (Life Technologies, Carlsbad, CA). For single cell RT-PCR, dispersed single RPE cells and retinal cells were placed on 12 mm #1 round coverslips and left to settle on the Nikon FN1 stage. About 2–5 cells (1 cell at a time) were picked using the recording pipette by applying negative pressure, and then expelled into a PCR tube containing RNA*later*® (AMBION, Austin, TX) by applying positive pressure. A single freeze-thaw cycle was used to disrupt the cell membrane in order to isolate the total RNA. mRNA was also isolated from a single RPE (pigmented cell) or retinal cell (bipolar or Muller glia). cDNA was synthesized using SuperScript III First-Strand Synthesis System (Life Technologies, Carlsbad, CA) following the manufacturer’s protocol. cDNA thus obtained was used for PCR using MyTaq HS Mix (Bioline USA Inc., Taunton, MA). One µl each of external forward and reverse primers (20 µM) were added to 25 µl of MyTaq HS mix. The volume of the reaction mix was brought to 50 µl with RNase-free water. Denaturing, annealing, and extension began with one cycle at 95 °C for 5 min, followed by 35 cycles at 94 °C for 30 s, 55 °C for 30 s, 72 °C for 30 s, and final extension at 72 °C for 10 min, respectively. The PCR product was further amplified for 25 cycles in the presence of internal primers using the same conditions as above. The PCR products were resolved using a 2% agarose gel (BIO-41026; Bioline, Taunton, MA, USA), and documented using the FOTO Dual Trans-illuminator (FOTODYNE, Hartland, WI, USA). All PCR primers are listed in Table 1.

**Table 1:** List of primers used for single cell RT-PCR.

Primers used for single cell PCR External Primers		
*Kcnj13*	Forward	5′-CTG GCT GAG ATG AAT GGT G-3′
	Reverse	5′-GTG AGC TAC TGC TGT G-3′
Gapdh	Forward	5′-TGA CAT CAA GAA GGT GA-3′
	Reverse	5′-CTC CTG TTA TGG GGG TC-3′
Internal Primers		
*Kcnj13*	Forward	5′-CTG GCT GAG ATG AAT GGT G-3′
	Reverse	5′-CTG TGA TAA AAG CCT CTA GCA-3′
Gapdh	Forward	5′-CAT TGC TCT CAA TGA CAA CTT T-3′
	Reverse	5′-GTG GTC CAG GGT TTC TTA CT-3′
Primers used to detect Kcnj13 post shRNA treatment		
*Kcnj13*	Forward	5′-CTG CGA TGG ACA GCA GTA AT-3′
	Reverse	5′-GTC CAC ACT GGT CTG GTA AAG-3′
*β*-actin	Forward	5′-GCT GTG CTA TGT TGC TCT-3′
	Reverse	5′-CTG TGT TGG CAT AGA GGT C-3′

### Immunohistochemistry

Enucleated eyes from the sacrificed mice were rinsed twice with PBS, a puncture was made at ora serrata with a 28 gauge needle and the eyes were immediately immersed in 4% paraformaldehyde (PFA) in PBS for 1 h. The fixed eyes were rinsed with PBS and cryoprotected using a sucrose gradient (10% sucrose × 1 h, 20% sucrose × 4 h, and finally by 30% sucrose overnight). The eyes were frozen with optical cutting temperature compound (Fisher Scientific) and 20 µm frozen sections were mounted on slides and stored at −80 °C. The frozen sections were rehydrated with PBS × 10 min and incubated with blocking solution containing 5% goat serum, 2% BSA and 0.3% Triton X-100 for 1 h. Primary antibodies were prepared in a 1:3 dilution of blocking solution containing antibody specific to Kir7.1 pre-labelled with ATTO-488 (1:50, Alomone labs, Jerusalem, Israel) and ezrin (1:200, Cell Signaling Technology, Danvers, MA). Alexa 594 anti-rabbit (1:1000, Molecular probes, Eugene, OR) was used as the secondary antibody for ezrin and was incubated for 1 h with 1:1000 DAPI. The sections were imaged with NIS-Elements using a Nikon C2 confocal microscope (Nikon Instruments Inc., Mellville, NY). We used 405, 488, and 561 nm Diode Lasers respectively for blue, green and red excitation and images, which were captured by Low Noise PMT C2 detectors in a Plan Apo VC 20X/0.75, 1 mm WD lens.

### Protein Isolation and Western Blotting

The posterior eye cup which includes both the RPE and the retina, was obtained as described above. The separated RPE and retina tissues were homogenized in Radioimmunoprecipitation assay buffer (RIPA, 100 mg tissue/ml) for protein isolation, incubated for 30 min in RIPA buffer on ice, then the homogenate was centrifuged at 16,000 g for 10 min. Protein concentration was determined using the BCA protein assay kit (Thermo Scientific). The protein lysate was incubated with Laemmli buffer (Bio-Rad, Herculus, CA) at 70 °C for 10 min. Thirty µg of protein from either RPE or retina was loaded in each well for SDS-PAGE. The protein samples were transferred to PVDF membrane and immersed in Odyssey blocking buffer^TM^ (LI-COR Biosciences) containing 0.1% Tween-20 (Sigma-Aldrich, St. Louis, MO) for 4 h. The membrane was incubated with primary antibodies for Kir7.1 (rabbit polyclonal raised using antigenic peptide from aa region 200–250, Osenses Pty Ltd, Keswick, Australia) and β-actin (mouse monoclonal, Cell Signaling, Danvers, MA) in blocking buffer at 4 °C overnight. After washing 4–5 min with TBS containing 0.2% Tween 20 (TBS-T), the membrane was incubated in anti- rabbit IgG conjugated to IR dye 800CW and anti-mouse IgG conjugated to IR dye 680RD secondary antibody for 1 h. After a final wash of the membrane with TBS-T, the membrane was scanned using two different channels of the Odyssey IR imager, and the results were analyzed using Image Studio (both from LI-COR Biosciences, Lincoln, NE).

### Electroretinography

The HMsERG system (Ocuscience Inc., Henderson, NV) was used for ERG recordings. A drop of 2% hypromellose (GONIOVISC, HUB Pharmaceuticals, LLC, Rancho Cucamonga, CA) solution was placed on the cornea to keep it moistened and to provide an electrical contact with the ERG electrode. The mice were placed under the 76 mm diameter Ganzfeld dome to assure uniform illumination of the eyes. The eyes were exposed to a sequential increment of flash intensities (0.1 to 10 cd.s/m^2^) for 400 ms with a 2 s interval between each flash. The data were analyzed using ERGVIEW (Ocuscience Inc., Henderson, NV) and Origin 9.1 (OriginLab Corp., Northampton, MA).

### *Ex vivo* ERG

We also used HMsERG system for *ex vivo* retina recording^51^. Retina was separated under complete darkness under a dissection microscope fitted with infrared camera (NVM14, ATN Corporation, San Francisco, CA) and infrared ring light (Advanced illumination, Rochester, VT). A quarter of retina with ganglion cell down was placed on a 3 mm nitrocellulose membrane (Ocuscience Inc., Henderson, NV) and transferred to *ex vivo* ERG tissue support system (HMsERG, Ocuscience Inc., Henderson, NV). Using our HMsERG mini Ganzfeld, we stimulated tissue with 100 mcd.s/m^2^ light flash during 5 ms for a 400 ms acquisition of ERG wave form. Retina was continuously perfused with Ame’s medium (SIGMA-Aldrich, St. Louis, MO) and temperature was controlled with an in-line heating system (PTC03, Scientific Systems Design Inc., Ontario, Canada). Drugs were directly dissolved in Ame’s medium. Data was stored for off line analysis in the ERGViEW and Origin9.1 software.

### Cell culture and transfection

Chinese Hamster Ovary (CHO-K1) cells grown in F-12 medium supplemented with 5% FBS and 1% Pen-Strep were used to transfect DNA, resulting in over-expression of the protein. 1 × 10^6^ cells grown in a 35mm culture dish were transfected with 2 µl of DNA using TransIT-LT1 (Mirus Bio, Madison, WI) reagent and were cultured at 37 °C in a 95% O_2_ −5% CO_2_ incubator.

### Optical Coherence Tomography (OCT) imaging

OCT is a non- invasive technique which is used to scan the retinal image and provide information on the health of the retina. The mouse was anesthetized and the pupil was dilated as explained above. The anesthetized mouse was immobilized on a 37 °C platform to assure thermostability and the eyes were hydrated with 0.5% carboxymethylcellulose sodium (Refresh Plus®, Allergan, Irvine, CA). OCT was performed on eyes injected with either saline as a control, or shRNA against Kir7.1, using Spectralis® OCT (Heidelberg Engineering, Heidelberg, Germany).

### Patch clamping

The whole-cell patch clamp configuration was used to measure Kir7.1 channel current from freshly isolated single RPE cells from mouse eye or transiently transfected CHO-K1 cells, as described previously^43^. Membrane potentials were amplified by Axopatch 200B (Molecular Devices, Sunnyvale, CA, USA) and command pulse was applied using pClamp software (version 9.2, Molecular Devices). Results were analyzed using Clampfit program (Molecular Devices) and Origin 9.1 (Origin Lab Corp., Northampton, MA).

### Statistical Analysis

Data are shown as mean ± SE. Student’s *t*-test was used for the statistical analysis. *P* values less than 0.05 were considered significant. Statistical analyses and graphs were plotted using Origin 9.1 (Origin Lab Corp., Northampton, MA).

## Electronic supplementary material

Supplementary information

Supplemental figures
