# Supplementary material for: Abnormal Electroretinogram after Kir7.1 Channel Suppression Suggests Role in Retinal Electrophysiology
Source: Sci Rep. 2017 Sep 6;7:10651. doi: 10.1038/s41598-017-11034-1 (PMC5587531; doi:10.1038/s41598-017-11034-1)
Supplement: Supplementary file 2 — Supplemental figures [file 41598_2017_11034_MOESM2_ESM.pdf]

Abnormal Electroretinogram after Kir7.1 Channel Suppression Suggests Role in Retinal Electrophysiology

Pawan K. Shahi, Xinling Liu, Bryce Aul, Andrea Moyer, Akshita Pattnaik, Jerod Denton,

De-Ann M. Pillers, Bikash R. Pattnaik#

#***Corresponding author:***

Bikash R. Pattnaik, PhD

Division of Neonatology

Department of Pediatrics

University of Wisconsin School of Medicine and Public Health

SMI-112, 1300 University Avenue

Madison, WI 53706, USA

Tel: 608 265 9486

E-mail: pattnaik@wisc.edu

Material and Method:

Histology: The eyes from the mice were enucleated and were fixed using 4% paraformaldehyde overnight and subsequently transferred to 30% sucrose solution until immersed completely. The eyeball was then frozen in OCT and processed for 10µm sections on slide. The tissue was then used for hematoxylin and eosin (H & E) staining to observe the variation in the morphology. H & E staining was performed following the standard protocol. In brief, the tissue section on the slide was rehydrated using PBS and covered with hematoxylin for a minute. The slide was then rinsed with water for 1 minute. The tissue was then stained with 1% eosin Y solution for 30 sec. Tissue was then immersed in 95% alcohol for 30 sec twice and immersed in the absolute alcohol for a minute. Finally, the slide was immersed in the xylene and using 40 X objective digital images were captured under bright field. For off line analysis, 25 μm^2 boxes were selected and ONL nuclei was counted.

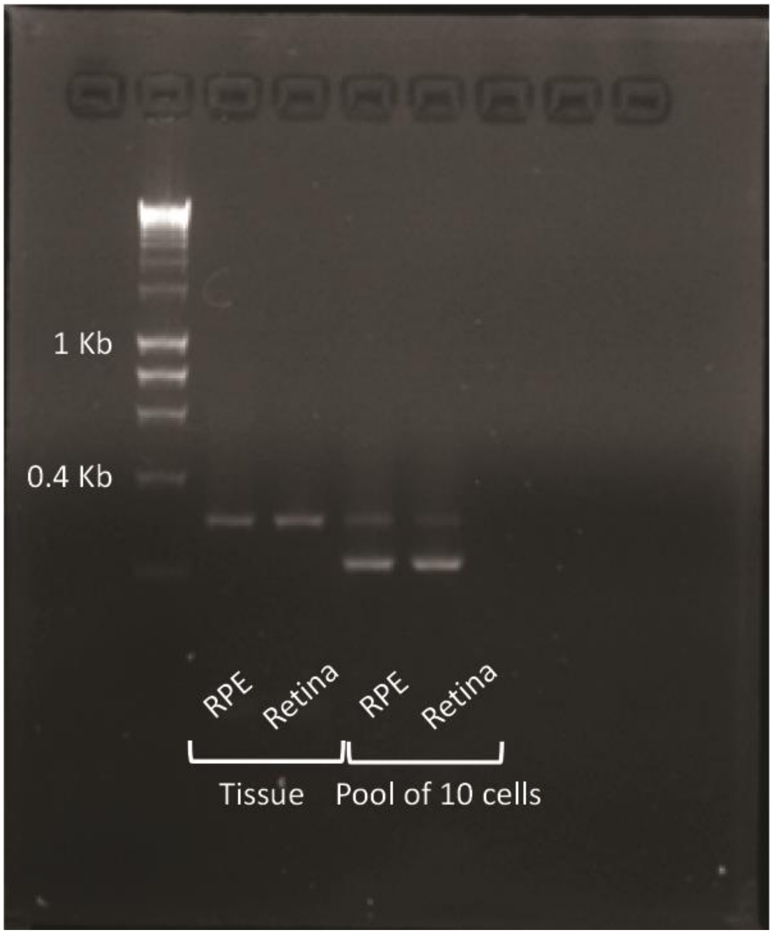

Supplemental Figure 1A. Full agarose gel image of Fig. 1A, mice tissue and cell expression of Kcnj13.

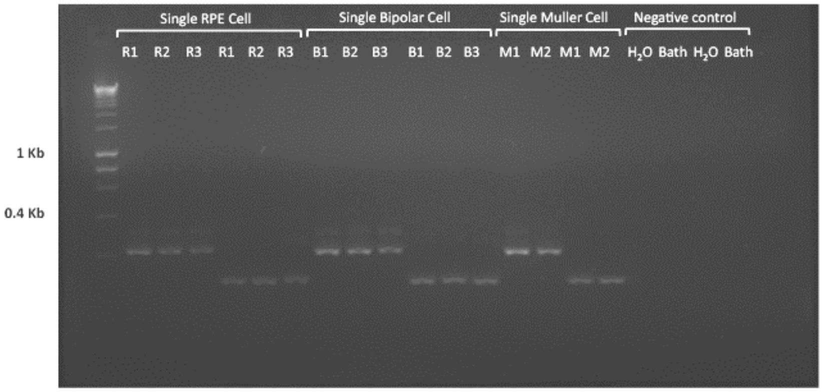

Supplemental Figure 1C. Full agarose gel image from individual cells for Kcnj13 expression shown in Fig. 1C.

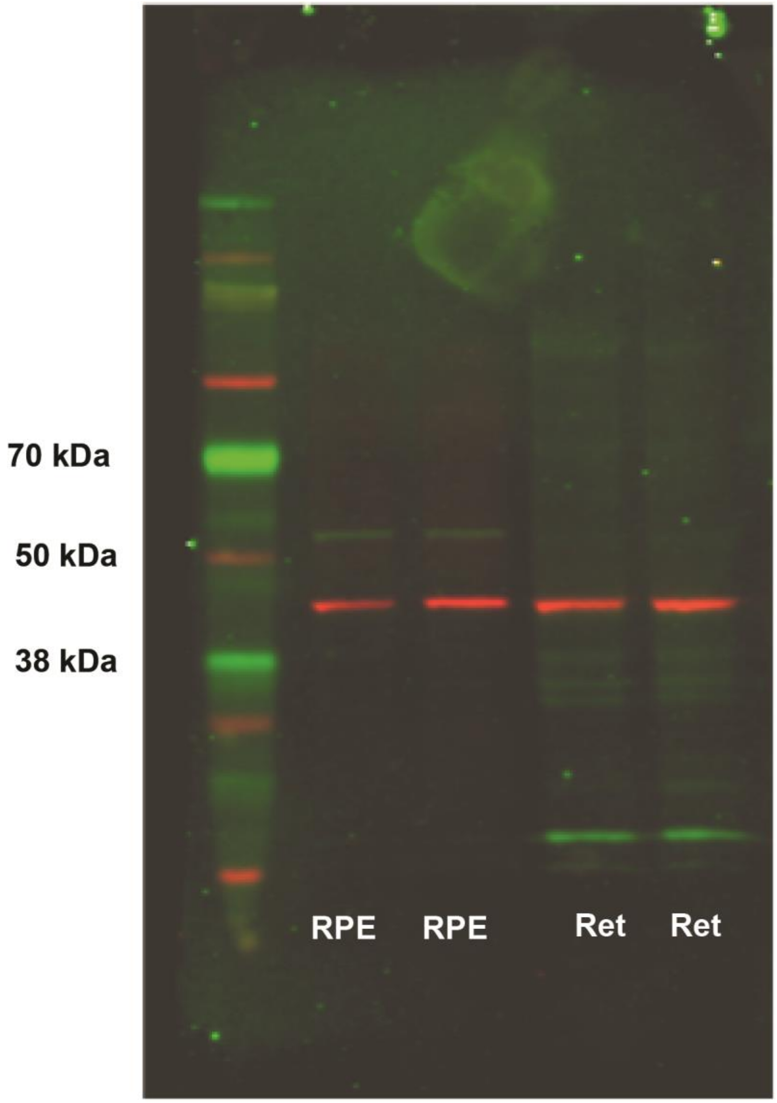

Supplemental Figure 2A. Full Western blot gel image example as in Fig. 2A.

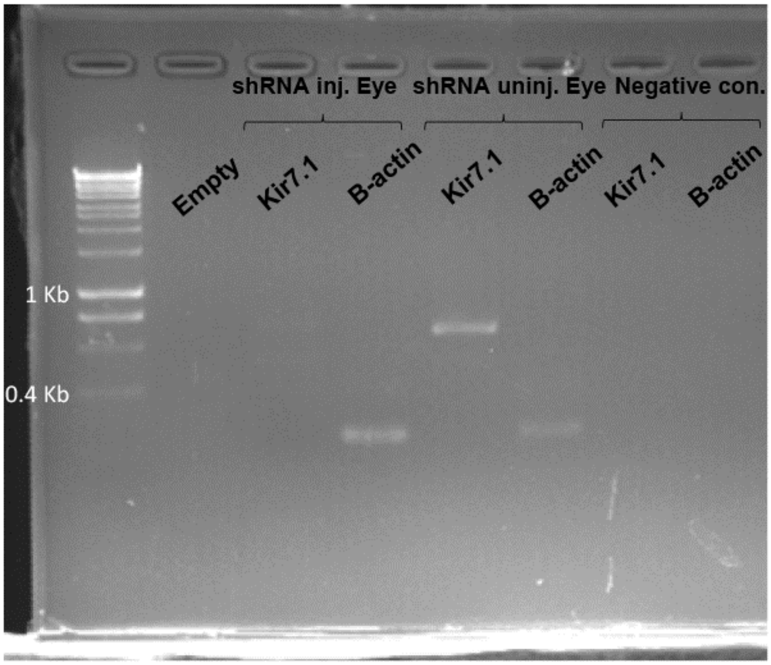

Supplemental Figure 3A. Full agarose gel image of mice Kcnj13 expression including negative control.

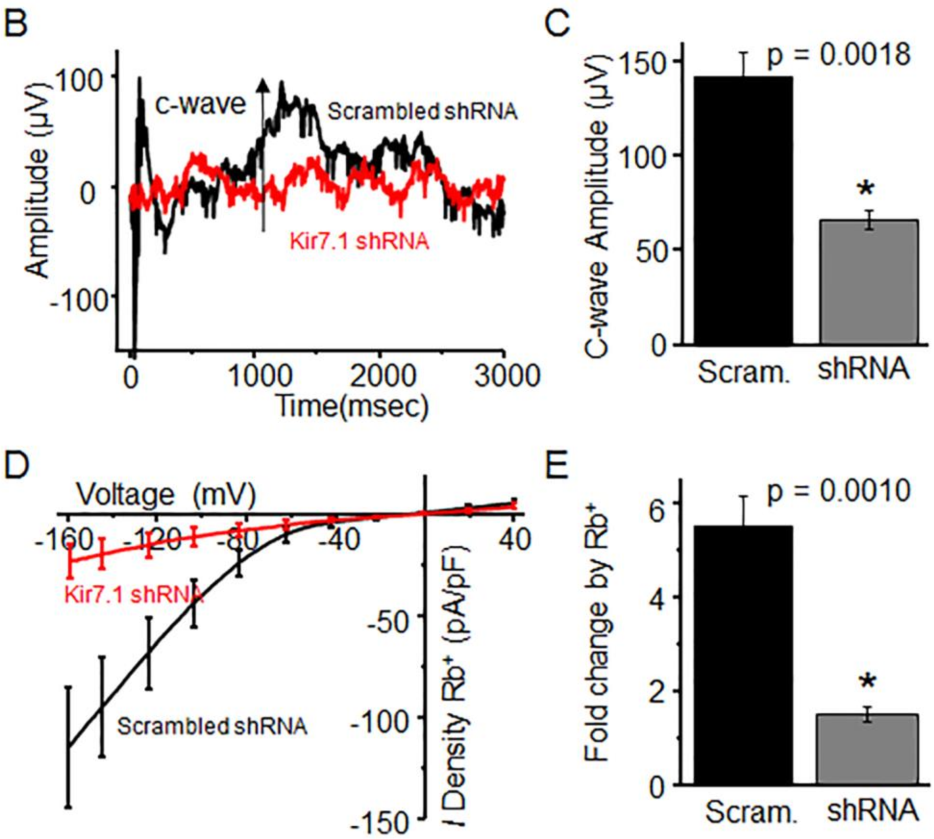

Supplemental Figure 3B-E. ERG was performed and c-wave was recorded from both scrambled shRNA and Kir7.1 shRNA injected mice (representative traces shown in B). Upward arrow indicates the measure of c-wave. The amplitude of c-wave in the scrambled shRNA injected eyes were unaffected whereas the reduction in the amplitude of c-wave in Kir7.1 shRNA injected eyes was distinct. (C) Average c-wave amplitude shows significant reduction in c-wave after silencing Kir7.1 channel. Data are mean ± SEM (n = at least 4 observations, P = 0.0018). (D) Functional assessment of the Kir7.1 channel from the isolated RPE cell from mice were performed from both scrambled shRNA (black trace) and Kir7.1 shRNA (red trace) injected eye after 14 days using the whole-cell patch clamp technique. No fold increment in Rb+ current through Kir7.1 channel conductance was observed after knocking out Kir7.1 by shRNA but not scrambled shRNA treated eye. (E) Average fold increase in Rb+ current. Data indicated as mean ± SEM (n = 5-7 cells/group, P = 0.0010).

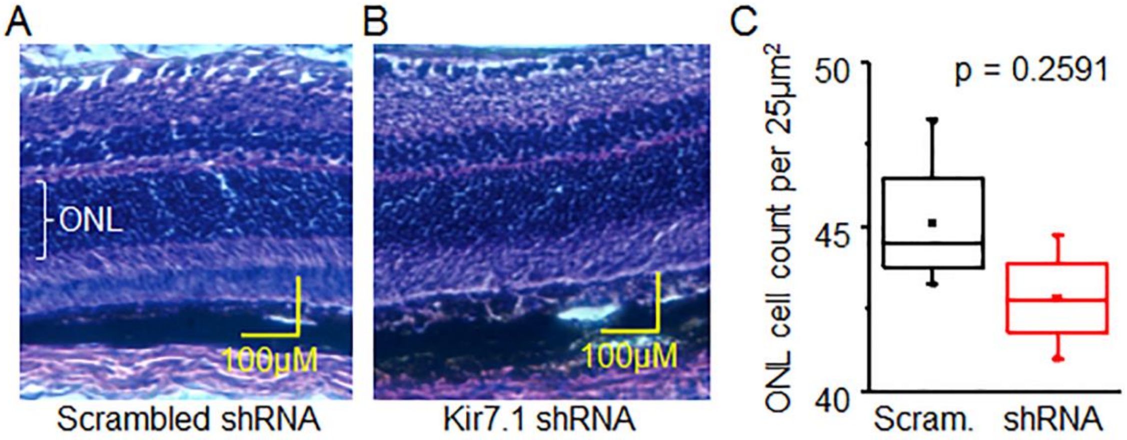

Supplemental Figure 5A-C. Effect of Kir7.1 silencing on retina morphology. Hematoxylin and eosin stain of the retinal section from the scrambled shRNA (A) and Kir7.1 shRNA injected (B) eyes post 14 days of injection (40X objective magnification) shows not much difference in the appearance and the morphology of different layers. Scale bar is as indicated and ONL is defined. (C) Average ONL cell count per 25 μm^2 from different regions between the scrambled shRNA and Kir7.1 shRNA injected eyes also showed no significant difference ($n = 5$, $P = 0.259$).

Supplementary Video. Confocal video reconstruction from Z-stack acquisition showing Ezrin (red) and Kir7.1 (green) expression in mouse eye section at 200X magnification. Z-section step size used was 0.82 µm per section and pixel size measured 0.38 µm. Image was acquired using a Nikon C2 confocal microscope.
